# Perspectives in melanoma: meeting report from the Melanoma Bridge (November 29th–1 December 1st, 2018, Naples, Italy)

**DOI:** 10.1186/s12967-019-1979-z

**Published:** 2019-07-22

**Authors:** Paolo A. Ascierto, Sanjiv S. Agarwala, Gerardo Botti, Alfredo Budillon, Michael A. Davies, Reinhard Dummer, Marc Ernstoff, Soldano Ferrone, Silvia Formenti, Thomas F. Gajewski, Claus Garbe, Omid Hamid, Roger S. Lo, Jason J. Luke, Oliver Michielin, Giuseppe Palmieri, Laurence Zitvogel, Francesco M. Marincola, Giuseppe Masucci, Corrado Caracò, Magdalena Thurin, Igor Puzanov

**Affiliations:** 1 Unit of Melanoma, Cancer Immunotherapy and Innovative Therapy, Istituto Nazionale Tumori IRCCS Fondazione “G. Pascale”, Via Mariano Semmola, 80131 Naples, Italy; 2 Medical Oncology and Hematology, St. Luke’s University Hospital and Temple University, Bethlehem, PA USA; 3 Istituto Nazionale Tumori-IRCCS Fondazione “G. Pascale”, Naples, Italy; 4 Experimental Pharmacology Unit, Department of Translational Research, Istituto Nazionale Tumori-IRCCS Fondazione “G. Pascale”, Naples, Italy; 5 Department of Melanoma Medical Oncology, Department of Systems Biology, University of Texas MD Anderson Cancer Center, Houston, TX USA; 6 Department of Dermatology, University of Zurich Hospital, Zurich, Switzerland; 7 Roswell Park Comprehensive Cancer Center, Buffalo, NY USA; 8 Massachusetts General Hospital, Harvard Medical School, Boston, MA USA; 9 Weill Cornell Medical College, New York Presbyterian Hospital, New York, NY USA; 10 Department of Pathology and Department of Medicine, Section of Hematology/Oncology, The University of Chicago Medicine, Chicago, IL USA; 11 Division of Dermatologic Oncology, Department of Dermatology, Eberhard Karls University, Tuebingen, Germany; 12 The Angeles Clinic, Experimental Therapeutics Cedars Sinai Foundation, Los Angeles, CA USA; 13 Jonsson Comprehensive Cancer Center, David Geffen School of Medicine at UCLA, Los Angeles, CA USA; 14 University of Chicago Medical Center, Chicago, IL USA; 15 Oncology Department, UNIL-CHUV, Lausanne, Switzerland; 16 Unit of Cancer Genetics, Institute of Biomolecular Chemistry, National Research Council, Sassari, Italy; 17 Institut de Cancérologie, Gustave Roussy Cancer Campus, Villejuif, Paris, France; 18 Refuge Biotechnologies, Menlo Park, CA USA; 19 Department of Oncology-Pathology, Karolinska Institute, Stockholm, Sweden; 20 Division of Surgery of Melanoma and Skin Cancer, Istituto Nazionale Tumori-IRCCS Fondazione “G. Pascale”, Naples, Italy; 21 Cancer Diagnosis Program, Division of Cancer Treatment and Diagnosis, NCI, 9609 Medical Center Drive, Bethesda, MD 20892-7420 USA; 22 Department of Medicine, Roswell Park Comprehensive Cancer Center, Buffalo, NY USA

**Keywords:** Melanoma, Immunotherapy, Anti-PD-1, Anti-CTLA-4, Target therapy, Biomarkers, BRAF inhibitor, MEK inhibitor, Adjuvant, Neoadjuvant, Combination strategies

## Abstract

Diagnosis of melanocytic lesions, correct prognostication of patients, selection of appropriate adjuvant and systemic therapies, and prediction of response to a given therapy remain very real challenges in melanoma. Recent studies have shown that immune checkpoint blockade that represents a forefront in cancer therapy, provide responses but they are not universal. Improved understanding of the tumor microenvironment, tumor immunity and response to therapy has prompted extensive translational and clinical research in melanoma. Development of novel biomarker platforms may help to improve diagnostics and predictive accuracy for selection of patients for specific treatment. There is a growing evidence that genomic and immune features of pre-treatment tumor biopsies may correlate with response in patients with melanoma and other cancers they have yet to be fully characterized and implemented clinically. For example, advancements in sequencing and the understanding of the tumor microenvironment in melanoma have led to the use of genome sequencing and gene expression for development of multi-marker assays that show association with inflammatory state of the tumor and potential to predict response to immunotherapy. As such, melanoma serves as a model system for understanding cancer immunity and patient response to immunotherapy, either alone or in combination with other treatment modalities. Overall, the aim for the translational and clinical studies is to achieve incremental improvements through the development and identification of optimal treatment regimens, which increasingly involve doublet as well as triplet combinations, as well as through development of biomarkers to improve immune response. These and other topics in the management of melanoma were the focus of discussions at the fourth Melanoma Bridge meeting (November 29th–December 1st, 2018, Naples, Italy), which is summarised in this report.

## Introduction

Significant improvements in melanoma diagnosis, prognosis and treatment have been achieved over the past decade. Improved insight into the genetic evolution of melanomas from their cells of origin through precursor lesion offers opportunities for improved diagnosis, earlier detection of lesions at increased risk of progression and selective intervention at an earlier stage. Recently, new therapeutic targets have emerged from the studies of the genetic profiling of melanomas through the identification of genetic mutations involved in the pathogenesis and malignant transformation of the melanocytes. Alterations of the RAS/RAF/MEK/ERK signalling cascade are considered drivers in majority of cutaneous melanomas development. NF1 is a tumor suppressor gene mutated in 10–15% of melanoma cases and is the third most frequently mutated gene in melanoma. KIT is an oncogenic driver associated with acral melanoma, and both are involved in proliferation and survival through the PI3K/AKT and the RAS/RAF/MEK/ERK pathways. Indeed, selective RAF and MEK and the PI3K)/AKT kinase pathways inhibitors (vemurafenib and dabrafenib) alone and/or in combination with MEK inhibitors (cobimetinib and trametinib) have shown promising results in clinical trials. For targeted therapy thus, it is possible to select the patients who will benefit from these treatments, based on the mutational profile of the tumor. Only patients with tumours harbouring BRAF mutations should undergo treatment with a BRAF inhibitor, and patients with known RAS-mutant should not receive this treatment. Several studies have indicated that BRAFV600E detection through ctDNA prior to the commencement of treatment is predictive of the response to BRAF kinase inhibitors, and that high basal ctDNA levels are associated with a lower response rate and progression-free survival. However, the clinical benefit of these therapies is limited, due to the rapid development of resistance through multiple mechanisms. Combination therapies seem to be an adequate strategy for melanoma patients, to overcome the resistance. Targeting signalling effectors downstream of driver oncogenes is a valid strategy to overcome resistance to BRAF inhibitors. MEK is a downstream target of BRAF and MEK inhibitors showed activity in NRAS-mutant melanomas.

Complex interactions between the tumor and the immune system play a role in melanoma development and metastatic spread to distant sites. Tissue infiltrating lymphocytes (TILs) recognize tumor-specific antigens, becoming activated and then proliferate and differentiate, acquiring the capacity to destroy cells that express tumor-specific antigens. In addition, to the stimulatory and inhibitory signalling pathways that limit T-cell antitumoral responses, cancer cells can escape T-cell detection, as usually they do express PD-L1 molecule.

Immunotherapy appears to be a promising treatment option for patients with advanced stage malignant melanomas, when compared to previous standard therapies, showing complete responses in selected patients. Major advances have been made in the treatment of metastatic melanoma using immune checkpoint blockade, with the FDA approval of numerous therapeutic regimens within the past several years and many more being studied in clinical trials. Treatment with immune checkpoint inhibitors such as monoclonal antibodies targeting cytotoxic T-lymphocyte-associated antigen 4 (CTLA-4) and programmed cell death protein 1 (PD-1) is associated with significant response rates, and many are durable. Several clinical trials are ongoing, using nivolumab and pembrolizumab in monotherapy or in combination with chemotherapy, radiotherapy, other immunotherapies, and targeted therapies are ongoing. Currently, the combination of two different immune checkpoint inhibitors or the combination of anti-PD1/anti-CTLA-4 with targeted therapy showed a clear benefit. However, most patients do not respond to these regimens as monotherapy, and some patients develop significant toxicity, particularly when these approaches are combined. There is a critical need to identify mechanisms of therapeutic resistance and adverse events in response to immune checkpoint inhibitors. There is emerging evidence that somatic mutations in antigen processing and presentation mechanism as well as up-regulation of genes involved in cell adhesion, angiogenesis, and extracellular matrix remodelling may contribute to immune escape in cancer. In addition, tumor-intrinsic oncogenic signals related to the WNT/β-catenin signalling pathway may mediate cancer immunity.

The immunogenic tumor microenvironment (TME), with mediators and cellular effectors of inflammation, influences the success of immunotherapies. The increasing understanding of the biological determinants of melanoma evolution and their potential integration in the management of melanoma patients may lead to an improved diagnosis, patient risk determination and potential for stratification for treatment. In recent years, improved knowledge of the pathophysiology and the role of the immune system in tumor evolution have led to the development and approval of biomarker(s) correlating with response to immunotherapy.

Increased tumor PD-L1 expression assessed by PD-L1 antibody using immunohistochemistry (IHC) and graded by the standardized scoring system is currently the only FDA approved and commercially available predictive biomarker in melanoma. At this point, PD-L1 expression on tumor specimens is not a candidate for pan-cancer marker for PD-1 inhibitor treatment response, due to the heterogeneous results obtained from clinical trials. For example, responses were also seen in PD-L1 negative tumors, thus making use of PD-L1 expression controversial and not standard of care in melanoma. While several genomic and immune predictors of response have been reported based on analysis of pre-treatment tumor biopsies, these biomarkers are not very robust, and there is significant overlap between responders and non-responders to therapy for the markers tested. Genomic studies exploring predictors of outcome to immune checkpoint blockade in melanoma suggest that tumor-specific mutational load and neoantigen signature are significantly associated with clinical benefit and increased overall survival. High mutational burden seems to associate with response in some studies, and it could correctly stratify the two groups of patients by response and predict progression free and overall survival. Gene expression profiling of the interferon-γ related profile was shown to predict best overall response, progression free survival, and overall survival in patients with melanoma treated with pembrolizumab. Immunohistochemistry-based studies also support the notion that CD8+ and CD4+ cell densities in pre-treatment biopsies can predict response to immunotherapy. Specific gut microbiota compositions can also drive differential responses to immune checkpoint inhibitors.

In addition to identifying predictors of response to immune checkpoint blockade, there is growing interest in understanding the mechanistic differences between different forms of immune checkpoint blockade. Transcriptome and pathway analysis of T cells and monocytes from patients on either CTLA-4 or PD-1 blockade demonstrates distinct gene expression profile and immunologic effects between these forms of therapy. Whereas CTLA-4 blockade induces a proliferative signature in memory T cells, PD-1 blockade leads to changes in genes underlying cytotoxic functions and NK cell function. Studies in animal models also demonstrate differential effects of CTLA-4 and PD-1 blockade therapies on the transcriptional profiles of tumor-infiltrating CD8+ T cells.

Together, these data have shown potential in selecting patients who are more likely to respond to immunotherapy and are currently being further investigated in clinical trials using agents targeting PD1 pathway. However, cumulative evidence from these studies suggests that these biomarkers are not perfectly predictive, and better biomarkers are clearly needed to optimize therapeutic decisions. Integration of these molecular tests may provide more comprehensive insight into an individual tumor’s behaviour and ultimately guide difficult management decisions in melanoma patients. Considering the complex biological interactions among different tumor pathways and their interplay with the immune system, bioinformatics approaches are required to yield conclusive results that can be translate into clinically applicable assays.

These and other topics in the management of melanoma were the focus of discussions at the fourth Melanoma Bridge meeting (November 29th–December 1st, 2018, Naples, Italy), which is summarised in this report.

## Melanoma as a model system session

### Neoadjuvant and adjuvant therapy for melanoma

Melanoma provides many opportunities to intervene therapeutically, including neoadjuvant and adjuvant therapy. In the adjuvant setting, interferon (IFN)-α is still available but is associated with high toxicity and its use remains controversial. It has, however, largely been superseded as checkpoint inhibitors and targeted agents have moved from the metastatic to adjuvant setting. In the first trial, 5-year relapse-free survival (RFS) rate with high-dose ipilimumab was 40.8% versus 30.3% with placebo (hazard ratio [HR] for recurrence or death, 0.76; P < 0.001) [1]. The overall survival (OS) rate at 5 years was 65.4% in the ipilimumab group compared with 54.4% with placebo (HR for death, 0.72; P = 0.001). This is the only adjuvant trial of these new agents to date to have shown an OS benefit. Despite the improved treatment, toxicity problems with ipilimumab, including five deaths in the trial, prevents it to be widely adopted and it appears unlikely to play a role as adjuvant treatment. The US Intergroup E1609 trial compared adjuvant ipilimumab (3 or 10 mg/kg) versus high-dose IFNα-2b for resected high-risk melanoma and found significantly less toxicity with the lower versus higher ipilimumab dose [2]. An unplanned RFS showed no difference in RFS between the two ipilimumab doses. Low-dose ipilimumab is undergoing further evaluation in the adjuvant setting.

Anti-PD-1 therapy has shown greater efficacy and less toxicity than ipilimumab in the adjuvant setting. In a trial of patients who underwent complete resection of stage IIIB-IV melanoma, nivolumab was associated with significantly improved 1-year RFS versus ipilimumab (70.5% versus 60.8%, HR for disease recurrence or death, 0.65; P < 0.001) [3]. Improved RFS was also observed with adjuvant pembrolizumab, with a 1-year RFS rate of 75.4% versus 61.0% with placebo (HR for recurrence or death, 0.57; P < 0.001) reported in patients with stage IIIA-C disease [4].

Adjuvant targeted therapy has also been shown to be effective. In patients with completely resected stage III BRAF-mutated melanoma, 3-year RFS rate was 58% with combined dabrafenib plus trametinib versus 39% with placebo (HR for relapse or death, 0.47; P < 0.001) with early separation of curves [5]. Thus, three trials showing a consistent beneficial effect on RFS with adjuvant PD-1 inhibitor therapy became available for melanoma patients although OS data is largely still awaited (Table 1).

**Table 1:** Comparison of anti-PD-1 adjuvant trials

Data comparison in adjuvant melanoma trials						
Patient population	CM238 [60]		COMBI-AD [61]		KN054 [4]	
	Completely resected stage IIIB/C or IV melanoma		Completely resected, *BRAF*^V600E/K^-positive stage IIIA/B/C melanoma		Completely resected stage IIIA/B/C melanoma	
Treatment	Nivo	Lpi	Dab/Tram	Placebo	Pembro	Placebo
N	453	453	438	432	514	505
RFS HR	0.65 (97.56% CI 0.51–0.83), P < 0.0001		0.47 (95% CI 0.39–0.58), P < 0.001		0.57 (98.4% CI 0.43–0.74), P < 0.001	
1-year RFS rate (%)	*71*	*61*	*88*	*56*	*75*	*61*
18-months RFS rate (%)	66	53	N/A	N/A	71	53
3-years OS rate (%)	N/A	N/A	86	77	N/A	N/A
Grade 3–5 TRAEs (%)	*14*	46	*31*	5	*15*	3
DC rate due to AEs (%)	*10*	43	*26*	3	*14*	2

In italic values for 1-y RFS Rate (%), patients treated with nivolumab Grade 3-5 TREs (%) and patients treated with nivolumab DC Rate due to AEs (%)

Comparison of three trials showing a consistent beneficial effect on RFS with adjuvant PD-1 inhibitor therapy, although OS data is largely still awaited

Although there is no available head-to-head comparison data to demonstrate whether anti-PD-1 or targeted therapy might be better option for BRAF-mutated patients, some differences can be noted. Anti-PD-1 therapy is active irrespective of BRAF status and is effective in BRAF-mutant and wild-type patients. With immunotherapy, RFS curves appear to have a step in first 1–3 months, after which the slope of the curve is flatter than with targeted therapy. To date, there are only interim OS data for targeted therapy with no OS data for immunotherapy. RFS data are also more mature with targeted therapy. Discontinuation rate due to toxicity is higher with targeted therapy than immunotherapy (25% versus 6–8%); however, acute toxicity on targeted therapy can be resolved by stopping treatment whereas anti-PD-1-related toxicity may be severe and require intervention. Trials conducted to date have subtle differences in the disease stages of enrolled patients which may be a consideration in treatment choice. Ongoing studies are also investigating patients with high-risk stage II and stage IV disease. Adjuvant therapy should be considered standard of care in high-risk patients, although high-risk is not clearly defined. Patients with stage IIIa disease may have good survival without intervention.

A key question is whether more patients with stage III melanoma are being cured through adjuvant therapy. This is currently unclear but is being investigated in the KEYNOTE-054 trial, which will assess whether post-surgery therapy with pembrolizumab improves RFS compared to placebo for high-risk patients with melanoma. In part 1 of the trial, patients will receive pembrolizumab or placebo as post-surgery therapy for up to 1 year. During Part 2, patients in either arm with documented recurrence may receive optional re-treatment with pembrolizumab for up to 2 years or disease progression.

Neoadjuvant therapy provides the opportunity to treat early in the disease course when the immune system should still be intact. It may reduce tumor burden before surgery and help guide additional adjuvant therapy. Pathological complete response (pCR) may also be a surrogate for RFS and OS. This is an area of active research with many trials ongoing. In a phase II trial, neoadjuvant plus adjuvant dabrafenib and trametinib significantly improved event-free survival (EFS) versus standard of care (upfront surgery and consideration for adjuvant therapy) in patients with high-risk, clinical stage III-IV BRAF-mutated melanoma [6]. This trial was stopped early because of the very strong signal for better EFS in the neoadjuvant dabrafenib and trametinib arm. Trials are also investigating nivolumab plus ipilimumab in combination, with different dosing regimens being evaluated. In the OPACIN trial, neoadjuvant ipilimumab 3 mg/kg plus nivolumab 1 mg/kg resulted in a 78% pathological response rate with all responders relapse-free after 3 years of follow-up [7]. However, 90% of patients experienced grade 3/4 toxicities. Nivolumab alone was less toxic but with an inadequate response rate. The subsequent OPACIN-NEO trial has reported a similar response rate with neoadjuvant ipilimumab 1 mg/kg plus nivolumab 3 mg/kg but with more manageable toxicity [8]. Pathological response was correlated with RFS and a baseline IFN-α signature was identified as a possible biomarker for treatment outcome.

Neoadjuvant therapy is a scientifically appealing approach with good data, but whether it will ever become standard of care is more questionable.

#### Key points

- The use of interferon in the adjuvant setting has been superseded by checkpoint inhibitors and targeted agents that have moved from the metastatic to adjuvant setting.
- Anti-PD-1 therapy has shown greater efficacy and less toxicity than ipilimumab in the adjuvant setting.
- Adjuvant targeted therapy has also been shown to be effective in patients with completely resected BRAF-mutated melanoma, but toxicity is higher than with immunotherapy.
- Neoadjuvant therapy provides the opportunity to treat early in the disease course and is an area of active research interest with many trials ongoing.

### Biomarkers in immunotherapy of melanoma: an update

Biomarkers for immunotherapy response in melanoma are not yet well established. However, two potential biomarkers out of numerous candidate biomarkers that have been the subject of considerable investigation are tumor mutational burden (TMB) and circulating tumor DNA (ctDNA).

TMB is determined by whole exome sequencing (WES) after removal of germline DNA sequence variants to consider only somatic alterations. TMB is defined as the number of somatic, coding, base substitution, and indel mutations per megabase of genome examined. Targeted next generation sequencing (NGS) panels, which are already being used for oncogenic mutation detection (e.g. by Foundation Medicine [F1CDx] and Memorial Sloan Kettering Cancer Center [MSK-IMPACT] are now being validated against WES data. Both panels have been approved by the US Food and Drug Administration for targeted-DNA-sequencing panel for specific somatic mutations [9].

Numerous clinical trials since 2014 have included TMB as a potential biomarker. In a study of 35 patients with stage IV melanoma treated with combined nivolumab and ipilimumab, an NGS panel sequencing of 710 tumor-related genes was used to assess TMB. High TMB was associated with significantly better OS, melanoma-specific survival and response, while patients with progressive disease had lower TMB. Patients who had received targeted therapy prior to immunotherapy had less favourable outcomes. High TMB has also been shown to be an independent predictor of response to immunotherapy in a diverse array of other cancers, including non-small-cell lung cancer (NSCLC) [10].

Certain cancer types have higher rates of high TMB tumors, especially those affected by exogenous carcinogens including UV and tobacco, such as melanoma, other skin cancers and lung cancer. The underlying mechanism of the TMB being independent biomarker for immunotherapy, is increased number of neoantigens resulting from SNVs that provides a higher likelihood a tumor being recognized as foreign. For patients with low TMB, studies to assess whether targeted agents and combination treatments may be more effective than immunotherapy are needed.

Analysis of ctDNA may also be a potential biomarker for response to immunotherapy. In a study of 76 metastatic melanoma patients treated with anti-PD-1 agents, significantly higher responses were seen in patients with undetectable ctDNA at baseline or elevated ctDNA at baseline but undetectable within 12 weeks of therapy compared to patients with elevated ctDNA at baseline that remained elevated during treatment [11]. Better PFS and OS were reported in patients with undetectable ctDNA at baseline or after 12 weeks. In another trial, a significant reduction in ctDNA levels after 2 weeks of treatment relative to baseline was associated with a clinical response to anti-PD-1 therapy, while a persistent decrease in ctDNA was associated with a durable response [12]. Patients with undetectable baseline ctDNA also had significantly improved OS, although this effect was not observed for PFS. It is not yet clear whether decrease in ctDNA levels during therapy or baseline level may be more important as a prognostic factor.

#### Key points

- Two potential biomarkers for immunotherapy that are of interest are tumor mutational burden (TMB) and circulating tumor DNA (ctDNA).
- Certain cancer types, such as melanoma, have higher rates of high TMB tumors and high TMB is an independent predictor of response to immunotherapy.
- For patients with low TMB, studies to assess whether targeted agents may be more effective than immunotherapy are needed.
- Higher response to anti-PD-1 therapy has been reported in patients with undetectable ctDNA at baseline and in patients with significant reductions in ctDNA after starting treatment.
- It is not yet clear whether decrease in ctDNA levels during therapy or baseline level can serve as an important as a prognostic factor.

### Mechanisms of targeted therapy resistance: from BRAF to NRAS mutant melanoma

Distinct genetic subtypes of melanoma with mutations in BRAF or NRAS develop resistance to MAPK inhibitors via hyper-activation of and addiction to the MAPK pathway. The convergence of multiple resistance mechanisms that reactivate (on drug) and hyper-activate (off drug) the MAPK pathway is a significant problem, which is only starting to be addressed by the combination of type I RAF inhibitors and allosteric MEK inhibitors. Because resistance is still commonly observed in BRAF mutant melanoma treated with BRAF and MEK inhibitors, additional strategies are warranted.

It is noteworthy that MEK inhibitors display marginal activity against NRAS-mutant melanoma (NEMO trial), likely due to both immunologic and tumor cell-intrinsic resistance [13]. Importantly, activity of MEK inhibitor in this trial was mostly limited to patients with prior treatment and failure on immune checkpoint blockade therapy. Also supported by preclinical studies in multiple syngeneic tumor models, including those of BRAF, NRAS and NF1 murine melanoma, the activity of a MEK inhibitor combined with anti-PD-1 blockade in patients with BRAF wild-type melanoma that displays innate anti-PD-1 resistance was studied. If anti-PD-1 therapy, despite innate resistance, primes a subsequent MEK inhibitor response, this sequencing paradigm may be broadly applicable to MAPK pathway-addicted cancers.

MAPK inhibitor resistance in melanoma is quite robust in experimental NRAS mutant melanoma with acquired resistance to a MEK inhibitor. Augmentation of MAPK resistance to achieve regression of melanoma can be achieved by discontinuation of the MEK inhibitor followed by addition of a PARP inhibitor [14]. This synthetic lethality strategy approach was already proposed in the clinic.

Finally, RAF dimerization among distinct resistance mechanisms (involving MAPK-reactivation) implies that next-generation MAPK inhibitors, such as dimeric RAF inhibitors, might be useful combinatorial partners to MEK inhibitors across all MAPK-dependent subtypes of melanoma. The combination of a dimeric RAF inhibitor with an allosteric MEK inhibitor can be highly synergistic, with the former having little activity alone. How these two classes of MAPK inhibitor work synergistically given their promise to overcome MAPK inhibitor resistance in BRAF, NRAF melanoma is under investigations.

#### Key points

- The convergence of multiple resistance mechanisms that reactivate (on drug) and hyper-activate (off drug) the MAPK pathway is a significant problem in treatment of melanoma.
- MEK inhibitors display marginal activity against NRAS mutant melanoma, likely due to both immunologic and tumor cell-intrinsic resistance.
- Anti-PD-1 therapy, despite innate resistance, may prime a subsequent MEK inhibitor response, suggesting that such sequencing paradigm may be broadly applicable to MAPK pathway-addicted cancers.
- Another strategy to overcome MAPK inhibitor resistance in melanoma involves exploitation of the MAPK inhibitor addiction phenotype. Discontinuation of the MEK inhibitor followed by addition of a PARP inhibitor to achieve regression of melanoma could represent one of the approaches.
- Next-generation MAPK inhibitors, such as dimeric RAF inhibitors, might be useful combinatorial partners to a MEK inhibitor across all MAPK-dependent subtypes of melanoma.

### Clinical evidence emerging from radiation and immunotherapy

The pre-existing immune infiltrate in tumors drives prognosis and response to immune checkpoint blockade. Integration of ionizing radiation targeted at lesions may help improve response to immunotherapy, as initially demonstrated in a syngeneic mouse model of breast cancer in which radiation and cytotoxic T-lymphocyte-associated antigen (CTLA)-4 blockade resulted in improved survival due to T cell-mediated control both of the irradiated tumor and lung metastases, as evidence for an abscopal effect [15]. The optimal dose and fractionation of radiation in combination with immunotherapy for the abscopal effect is yet unknown. Single dose 30 Gy radiation with anti CTLA-4 has been shown to preclude the abscopal effect and this has been attributed to the induction of DNA exonuclease Trex1 by radiation doses above 12–18 Gy per fraction in different cancer cells. Trex1 attenuates immunogenicity by degrading DNA that accumulates in the cytosol upon radiation [16]. Cytosolic DNA stimulates secretion of IFN-β by cancer cells following activation of the DNA sensor cGAS and its downstream effector STING. Repeated irradiation at doses that do not induce Trex1 amplifies IFN-β production, required for priming of CD8+ T cells that mediate the abscopal effect in the context of immune checkpoint blockade. These preclinical findings suggest that optimal effects of radiation and immune checkpoint blockade depend upon the dose per fraction of radiation used.

Consistently, the use of pembrolizumab in combination with classical large single doses of radiotherapy in patients with advanced solid tumors resulted in disappointing results, with an overall objective response rate (ORR) of 13% and no complete responses [17]. Interestingly, in another study, patients with metastatic NSCLC who received pembrolizumab and radiotherapy delivered in 3 fractions of 8 Gy had a significantly better response versus patients receiving pembrolizumab alone (41% vs 19%) [18]. These contrasting results provide preliminary evidence for a dose/fraction effect, that confirm the preclinical data.

Another question is whether radiotherapy and checkpoint blockade should be concurrent or sequential. In the PACIFIC trial, durvalumab after chemo radiotherapy significantly increased progression-free survival (PFS) irrespective of PD-ligand (L) 1 expression at baseline [19]. In another study in patients with advanced NSCLC, previous treatment with radiotherapy, even up to a year earlier, resulted in longer PFS and OS with pembrolizumab treatment than that in patients without previous radiotherapy [20].

Can radiation therapy in combination with immune checkpoint blockade convert an irradiated human cancer into an in situ vaccine? CTLA-4 blockade had previously failed to demonstrate significant efficacy alone or with chemotherapy in locally advanced and metastatic NSCLC. However, in a trial of 39 patients with chemo-resistant metastatic NSCLC the addition of focal radiation therapy to one metastasis during ipilimumab induced systemic anti-tumor T cells and achieved an 18% ORR, indicating that radiation had ‘repositioned’ CTLA-4 checkpoint inhibition in this disease setting [21]. There was no association between response and site of irradiation or previous PDL-1 tumor expression. Increased serum IFN-β post-radiation and early dynamic changes of blood T cell clones were the strongest predictors of response.

Radiotherapy plus immune checkpoint inhibition can generate neo-epitopes that impart a memory effect. Functional analysis in one responding patient showed the rapid in vivo expansion of CD8 T cells recognizing a neoantigen encoded in a gene that is upregulated by radiation and was mutated in that specific patient. This case supports the hypothesis that radiation-induced exposure of immunogenic mutations to the immune system explain the immunogenicity of radiation (that can clinically manifest as abscopal response). The gene that encoded this mutation in karyopherin A2 (KPNA2, also known as importin-α) is known to be upregulated by radiotherapy in human cancer cells in vitro and was also upregulated in vivo after radiation in a patient-derived xenograft NSCLC model.

#### Key points

- Ionizing radiation targeted at lesions may help improve response to subsequent immunotherapy.
- The optimal dose and fractionation of radiation in combination with immunotherapy for the abscopal effect is yet unknown.
- Preclinical evidence suggests that the optimal effects of radiation and immune checkpoint blockade depend upon the dose per fraction of radiation used.
- Radiotherapy plus immune checkpoint inhibition can generate neo-epitopes that impart a memory effect.

## Mechanisms of resistance and drivers of response session

### Targeted therapy and immunotherapy: rational for combination and future perspectives

Immunotherapy is slow-acting with the potential for long-term benefit while targeted therapy is fast-acting and promotes a rapid metabolic shutdown but with possible resistance. In the BRIM-7 study of cobimetinib and vemurafenib in BRAF-mutant melanoma, OS appeared to plateau at 4 years with an OS rate of 39% that was maintained at 5 years, suggestive of a subgroup of patients with prolonged survival [22]. In a pooled analysis of trials, baseline lactate dehydrogenase (LDH), ECOG performance status, disease burden, and gene signature appeared to be key determinants of survival outcomes in patients treated with cobimetinib plus vemurafenib [23]. In the most favourable subgroup (normal LDH and baseline sum of longest diameters of target lesions [SLD] ≤ 45 mm), 3-year OS rate was 53.3% with cobimetinib plus vemurafenib. The immune signature was enriched in favourable clinical prognostic subgroups, consistent with an immunosuppressive effect of oncogenic BRAF signalling on the tumor-host interaction and beneficial effects of MEK inhibition on response to immune checkpoint inhibitors. Baseline LDH and SLD were also predictive of PFS and OS in a 3-year pooled analysis of trials of dabrafenib and trametinib combination therapy [24].

The first evidence that lack of PD-L1 expression and presence of immune cell infiltration predict a better response in BRAF inhibitor-treated BRAFV600-mutant melanoma was reported by Massi et al. in 2015 [25]. However, patients with < 1% CD8+ T cells in the tumor centre or lower PD-L1 expression have been observed to derive clear benefit from vemurafenib. These data suggest an immune-action of tyrosine kinase inhibitors.

Three immune phenotypes point to disruption of specific steps of the cancer-immunity cycle. The immune desert phenotype involves non-inflamed tumors with little or no CD8+ T-cell infiltration, the immune-excluded phenotype involves non-inflamed tumors but with the presence of CD8+ T-cells residing solely in the periphery, and the immune-inflamed phenotype involves the presence of intratumoral CD8+ T-cell infiltrate. Each phenotype requires a different strategy to reinitiate the anti-tumor immune response. For immune desert tumors, the aim is to generate, release and deliver antigens, and enhance antigen presentation and T-cell priming. For immune-excluded tumors, the goal is to recruit T cells to the tumor and address the stromal barrier, while for already inflamed tumors, the goal is to invigorate T cells. Redirecting and engaging T cells is required for all tumor phenotypes. Blocking the MAPK signalling pathway results in changes in the TME, including increased melanoma antigen expression, decreased immunosuppressive cytokine production, increased CD8+ T-cell infiltration, increased T-cell clonality, increased PD-L1 expression and class I MHC upregulation. This may mean tumor cells are more visible and hence more susceptible to immunotherapy.

MEK inhibition may help to unleash a greater anti-tumor potential of PD-L1 inhibition via direct effects on T cells and the TME. For example, increases in CD8+ T cells have been observed following cobimetinib plus vemurafenib. Several trials combining BRAF and MEK inhibition with an anti-PD-1/PD-L1 agent are ongoing with promising results. These include combined atezolizumab plus vemurafenib and cobimetinib in BRAF V600-mutant metastatic melanoma, which has shown promising antitumor activity and a manageable safety profile in phase I data with phase II/III studies ongoing [26]. Another emerging combination approach to modulate tumor immunogenicity is based on the use of histone deacetylase (HDAC) inhibitors, epigenetic modifiers that exert this effect on cancer cells as well as on immune cells and components of the TME [27]. Several clinical trials of HDAC inhibitors in combination with immune checkpoint blockers are ongoing. For example, the efficacy and safety of the HDAC inhibitor entinostat and pembrolizumab in patients with melanoma progressing on or after PD-1/L1 blocking antibody, has recently been reported [28].

#### Key points

- Immunotherapy is slow-acting with the potential for long-term benefit while targeted therapy is fast-acting and promotes a rapid metabolic shutdown but with possible resistance.
- The immune signature is enriched in prognostic subgroups with a favourable response to BRAF and MEK inhibition, consistent with an immunosuppressive effect of oncogenic BRAF signalling on the tumor-host interaction.
- Blocking the MAPK signalling pathway results in changes in the TME that may mean tumor cells are more visible and hence more susceptible to immunotherapy.
- Several trials combining with an anti-PD-1/PD-L1 agent are ongoing with promising results.

### Exosomes in melanoma

Tumors produce and release a variety of extracellular vesicles, the smallest (30–150 nm) of which are called exosomes. These tumor-derived exosomes (TEX) have recently emerged as carriers of numerous receptors, ligands and factors that modulate functions of recipient cells. Because of their endocytic origin and the protein/genetic cargo, which mimics that of parent tumor cells, TEX have emerged as potential circulating biomarkers for parent tumor cells. TEX also serve as regulatory elements, because they reprogram and alter the functions of recipient cells, carry numerous immunoinhibitory signals and down-regulate functions of effector immune cells. TEX also re-program immune cells and could serve as potential immune biomarkers.

To date, TEX have largely been studied as by-products of tumor cell lines maintained in cultures. Studies of plasma-derived exosomes have been challenging, and several limitations have been identified, including the lack of a rapid and cost-effective isolation method for exosomes in body fluids. However, isolation of intact, biologically active exosomes from cancer patients’ plasma or other body fluids is now possible by mini-size exclusion chromatography (mini-SEC) [29].

The monoclonal antibody 763.74 is specific for a peptide epitope of a tumor antigen expressed on melanoma cells and not generally detectable on normal cells, chondroitin sulphate peptidoglycan 4 (CSPG4) can be used to capture melanoma cell-derived exosomes. The CSPG4 epitope is present on > 80% of melanomas and is carried by exosomes produced by melanoma cells. CSPGs are key bioactive molecules that play a major role in tumor growth, migration and neo-angiogenesis.

Melanoma-derived exosomes (MTEX) were separated from non-tumor cell-derived exosomes and the protein cargo of both fractions were evaluated by quantitative flow cytometry [30]. Melanoma-associated antigens (CSPG4, TYRP2, Melan A, Gp100 and VLA4 were carried by MTEX, especially those from patients with advanced disease, but were not detectable in exosomes produced by normal cells. MTEX were enriched in FasL and TRAIL whereas non-MTEX were enriched in co-stimulatory proteins (CD40, OX40, OX40L). MTEX co-incubated with human primary T cells inhibit T-cell proliferation and MTEX co-incubated with primary activated CD8+ T cells induce apoptosis. Thus, isolated exosomes maintain their morphology and are functional. The ability to isolate functional MTEX and to assess for melanoma-associated antigens or other molecular or genetic markers of the tumor will facilitate testing of the role of MTEX as liquid biopsies in melanoma.

#### Key points

- Tumors produce and release a variety of extracellular vesicles, the smallest of which are exosomes, carriers of numerous receptors, ligands and factors that modulate functions of recipient cells.
- These tumor-derived exosomes (TEX) emerged as potential circulating biomarkers for parent tumor cells.
- Isolation of intact, biologically active exosomes from cancer patients’ plasma or other body fluids is now possible by mini-size exclusion chromatography.
- Melanoma-derived exosomes (MTEX) carry melanoma-associated antigens that are not detectable in exosomes produced by normal cells.
- The ability to isolate functional MTEX and to assess for melanoma-associated antigens or other molecular or genetic markers of the tumor will facilitate testing of the role of MTEX as liquid biopsies in melanoma.

### Intestinal ecosystem and immunity against melanoma

The relationship between the gut microbiome and host immunity raises the possibility that dysbiosis of the intestinal flora may influence the outcome of cancer immunotherapy. It has been shown that the anti-tumor efficacy of CTLA-4 blockade is supported by gut bacteria, with T cell responses specific for *Bacteroides thetaiotaomicron* or *B. fragilis* associated with improved efficacy [31]. It has also been shown that antibiotics inhibited the clinical benefit of anti-PD-1 or anti-PD-L1 treatment in patients with advanced cancer, significantly reducing PFS and OS [32]. Antibiotics were also associated with reduced clinical benefit from immune checkpoint inhibitors in patients with advanced renal cell carcinoma (RCC) or NSCLC [33].

**Fig. 1:**
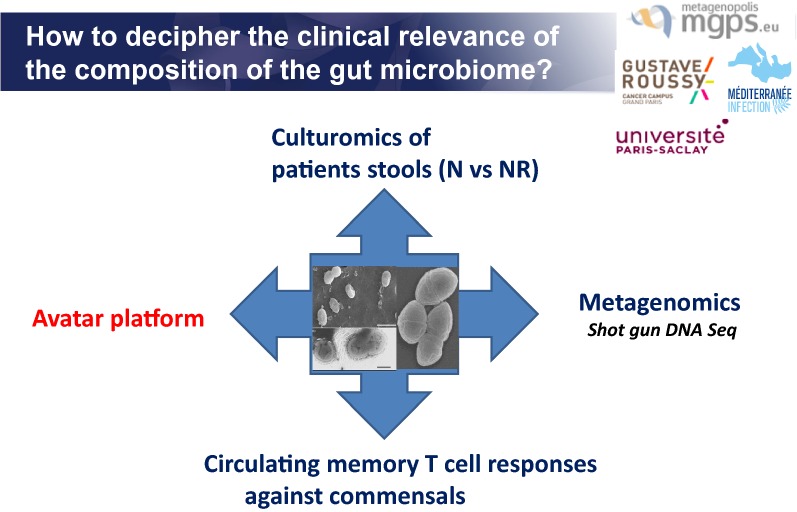
How to decipher the clinical relevance of the compositions of the gut microbiome? Various methods can be utilized to help better understand the clinical relevance of the composition of the gut microbiome

Various methods can be utilized to help better understand the clinical relevance of the composition of the gut microbiome (Fig. 1).

The transplantation of fecal microbiota (FMT) from cancer patients who responded to immune checkpoint blockade into germ-free or antibiotic-treated avatar mice ameliorated the anti-tumor effects of PD-1 blockade, unlike FMT from non-responding patients [32].

Shotgun, or untargeted, metagenomic sequencing, which uses genetic analyses of DNA from several types of cells, performed in two independent cohorts of patients with advanced RCC or NSCLC revealed that *Akkermansia muciniphila*, *Alistipes* spp. and *Ruminococcaceae* spp. were enriched in responders to checkpoint blockade.

*Akkermansia muciniphila* was validated as a predictor of best outcome in a second cohort of patients treated with anti-PD-1 antibodies. Circulating memory T cell responses against commensal bacteria were also investigated and showed that T cell responses against *A. muciniphila* and *Enterococcus hirae* were associated with longer survival during PD-1 blockade. Enhanced systemic and anti-tumor immunity was suggested in patients who responded to checkpoint inhibitors with a favourable gut microbiome and in germ-free mice receiving fecal transplants from responders. The classical method of stool culturomics also revealed that *E. hirae* were more frequent in faeces from patients with NSCLC who responded to anti-PD-1 treatment compared to non-responders. Restoration of anti-PD-1 antibody efficacy has been demonstrated using *A. muciniphila* across different tumor models using FMT from different patients. Random bacteria (*E. faecalis* or *B. nordii*) did not compensate the dysbiosis.

Studies to assess the role of the microbiome in melanoma are now ongoing. One trial is investigating concurrent FMT with pembrolizumab in patients with PD-1 resistant/refractory melanoma. Another study will involve altering the gut microbiota of melanoma patients scheduled to receive PD-1 blockade using FMT from patients who responded to anti-PD-1 therapy. In the future, microbiome modulation may become an important modality in cancer therapy.

#### Key points

- Dysbiosis of the intestinal flora may influence the outcome of cancer immunotherapy and it has been shown that the anti-tumor efficacy of CTLA-4 blockade is supported by gut bacteria, with T cell responses specific for *Bacteroides* spp. associated with improved efficacy.
- Antibiotics may inhibit the clinical benefit of anti-PD-1 or anti-PD-L1 treatment in patients with advanced cancer.
- Enhanced systemic and anti-tumor immunity was suggested in patients who responded to checkpoint inhibitors with a favourable gut microbiome and in germ-free mice receiving fecal transplants from responders.
- Studies to assess the role of the microbiome in melanoma are now ongoing with the hope that microbiome modulation may become an important modality in cancer therapy.

### Tumor and host factors regulating immunotherapy efficacy

The T cell-inflamed and non-inflamed TMEs represent two categories of immune escape. Whereas the T cell-inflamed tumor is characterized by chemokine expression increased CD8+ T cells and a type I IFN signature with immune escape via inhibitory pathways resulting in increased anti-PD-1 efficacy, non-inflamed tumors have a low inflammatory signature and absent intratumoral CD8+ T cells with immune escape via T cell exclusion, meaning anti-PD-1 efficacy is diminished. Activity of anti-PD-1 in multiple cancers is associated with a T cell-inflamed TME signature at baseline and the T cell-inflamed TME serves as an approximate predictive biomarker for response to anti-PD-1-based immunotherapies [34].

Although the presence of tumor-infiltrating lymphocytes (TILs) indicates an endogenous antitumor response, multiple immune regulatory pathways can subvert the effector phase and enable tumor escape. Negative regulatory pathways include extrinsic suppression mechanisms, but also a T cell-intrinsic dysfunctional state characterized by co-expression of the cell surface proteins lymphocyte-activating gene (LAG)-3 and 4-1BB. LAG-3 and 4-1BB+ TILs actively proliferate in situ but also actively apoptose, creating a self-defeating cycle. TIL apoptosis is associated with antigen-induced T-cell dysfunction, possibly due to the accumulation of DNA damage in dysfunctional TILs. A 4-1BB agonist antibody synergised with checkpoint blockade to decrease TIL apoptosis and enhance tumor control [35]. Mechanisms of human TIL apoptosis should be investigated to develop new potential therapeutic strategies for T cell-inflamed tumors.

Molecular mechanisms to explain the T cell-inflamed versus non-inflamed TMEs include somatic differences at the level of tumor cells e.g. the mutational landscape and antigenic repertoire and distinct oncogene pathways activated in different patients, germline genetic differences at the level of the host such as polymorphisms in immune regulatory genes, and environmental differences in commensal microbiota and the immunological exposure history of patients.

Multiple lines of evidence indicate a critical role for Batf3-lineage dendritic cells (DCs) at multiple levels in anti-tumor immunity and immunotherapy efficacy. The first oncogene pathway identified that mediates immune exclusion is the Wnt/β-catenin pathway. Tumor cell-intrinsic β-catenin activation prevents the host anti-tumor immune response by failure to recruit Batf3 DCs, upon which the recruitment of effector CD8+ T cells is dependent [36]. Batf3-lineage DCs are involved in both the priming phase and the effector phase of the anti-tumor immune response and there is a loss of efficacy of anti-PD-L1 upon depletion of DCs at the effector phase.

T cell exclusion from the TME mediated by β-catenin, PTEN loss, and other oncogenic events is prompting re-focused drug development. Activation of tumor-intrinsic WNT/β-catenin signalling is enriched in non-T cell-inflamed tumors and secondary recurrence in melanoma is associated with upregulated β-catenin and loss of immune signature. Secondary resistance to ipilimumab and nivolumab has also been associated with PTEN deletion.

Molecular mechanisms that mediate the presence or absence of the T cell-inflamed TME are being elucidated using parallel genomics platforms. In vitro screening for genetic alterations is identifying genes (MHC class I, IFN-γR2, Jak1) mediating resistance to T cell-mediated killing. Multidimensional ‘omics’ analyses are identifying individualized molecular correlates of response versus resistance in patients, including commensal microbiota and germline genetic variants.

#### Key points

- The T cell-inflamed and non-inflamed TME represents two categories of immune escape.
- Activity of anti-PD-1 is associated with a T cell-inflamed TME signature at baseline and the T cell-inflamed TME serves as an approximate predictive biomarker for response to anti-PD-1-based immunotherapies.
- Mechanisms of human TIL apoptosis should be investigated to develop new potential therapeutic strategies for T cell-inflamed tumors.
- Molecular mechanisms that mediate the presence or absence of the T cell-inflamed TME are being elucidated using parallel genomics platforms.

### Intratumoral immunotherapy a weapon beyond a “mass” destruction

There are many ongoing studies evaluating oncolytic intratumoral immunotherapy in combination with checkpoint inhibition. The already approved oncolytic virus talimogene laherparepvec (T-VEC) had a tolerable safety profile in combination with ipilimumab with improved efficacy versus either T-VEC or ipilimumab monotherapy in a phase Ib trial in patients with advanced melanoma [37]. In a phase II study, ORR was significantly higher with T-VEC plus ipilimumab versus ipilimumab alone (39% versus 18%) without additional safety concerns [38]. T-VEC is also now being assessed in combination with pembrolizumab in the phase III MASTERKEY-265 trial.

HF10 is a bioselected replication-competent oncolytic virus also derived from HSV-1 that demonstrated a favourable benefit/risk profile and encouraging anti-tumor activity with a best ORR of 41% when combined with ipilimumab in patients with stage IIIB–IV unresectable or metastatic melanoma [39]. A second oncolytic virus demonstrating promising clinical findings is coxsackievirus A21 (CVA21). An immunotherapeutic strain of CVA21 was assessed in combination with ipilimumab and showed clinical activity with a best ORR of 38% together with low adverse toxicity in patients previously treated with anti-PD-1 agents [40].

An intralesional agent, PV-10, which contains an injectable formulation of rose bengal disodium, is being assessed in combination with pembrolizumab. Interim results from a phase Ib trial indicated robust responses in target lesions, a 65% ORR with no unexpected toxicities [41].

Interleukin (IL)-12 augments the immune response but systemic IL-12 can cause significant toxicity. This can be avoided through electroporation which facilitates cell entry of plasmid IL-12 and results in high levels of IL-12 protein expression. Intratumoral plasmid IL-12 with electroporation increases TILs in both treated and untreated lesions. In patients treated with IL-12 electroporation and pembrolizumab, a 40% clinical response rate was observed and good tolerability [42].

The rationale for combining intralesional therapy with checkpoint blockade is the potential synergy from the immune stimulation effected after intralesional agents promote the release and presentation of tumor-derived antigens, thereby increasing TILs and turning tumors from an immunologically ‘cold’ to ‘hot’ status. A significant proportion of patients do not respond to immune checkpoint blockade. Identifying patients unlikely to respond is a challenge but the relative abundance of partially exhausted tumor-infiltrating CD8+ T cells has been shown to predict response to anti-PD-1 therapy [43]. Intralesional agents offer the potential to increase CD8+ T cells in the tumor, making them more responsive to immune checkpoint inhibition.

#### Key points

- Ongoing studies are evaluating oncolytic intratumoral immunotherapy in combination with checkpoint inhibition.
- Oncolytic virus demonstrating promising clinical findings include HF10, a bioselected replication-competent oncolytic virus derived from HSV-1, and coxsackievirus A21 (CVA21).
- An intralesional agent, PV-10, which contains an injectable formulation of rose bengal disodium, is being assessed in combination with pembrolizumab.
- Intratumoral plasmid IL-12 with electroporation has shown promising activity in patients and good tolerability.
- Intralesional therapy may promote the release and presentation of tumor-derived antigens, thereby turning tumors from an immunologically ‘cold’ to ‘hot’ status and so providing potential synergy with checkpoint blockade.

## Emerging strategies session

### Translational research in the therapeutic landscape for metastatic melanoma

One of the major technologies in translational research is NGS, with many studies having used WES or whole genome sequencing (WGS) on melanoma tumors, melanoma short term cultures or melanoma cell lines. However, a multitude of other cutting-edge technologies are also involved in translational research. These include single-cell mass cytometry/imaging mass cytometry for in-depth characterization of tumor and immune compartments at single cell level, with identification of cell subsets including phenotypic and functional features. Fast drug-testing measures ex vivo drug responses using automated microscopy, single cell image-analysis, and machine learning, while deep drug testing for molecular characterization of tumor cell response to drug treatment by analysing survival, proliferation, and differentiation pathways. Genomics and transcriptomics can infer tumor heterogeneity by single-cell and bulk RNA sequencing and single cell DNA sequencing to identify and quantify tumor sub-clones and normal cell types in the TME, including immune cell subtypes.

Some examples of the role of these techniques in translational research in melanoma are outlined below. Single-cell RNA sequencing of malignant cells isolated from BRAF-mutant patient-derived xenograft melanoma cohorts exposed to concurrent RAF/MEK inhibition resulted in the identification of distinct drug-tolerant transcriptional states, varying combinations of which co-occurred within minimal residual disease from patient-derived xenografts and biopsies of patients on treatment [44]. One of these exhibited a neural crest stem cell (NCSC) transcriptional program largely driven by the nuclear receptor RXRG. An RXR antagonist mitigated accumulation of NCSCs in minimal residual disease and delayed the development of resistance.

Analysis of somatic mutanomes and transcriptomes of pre-treatment melanoma biopsies identified a transcriptional signature (known as innate anti-PD-1 resistance, IPRES), indicating concurrent up-expression of genes involved in the regulation of mesenchymal transition, cell adhesion, extracellular matrix remodelling, angiogenesis, and wound healing [45]. MAPK inhibitor therapy induces similar signatures in melanoma, suggesting that a non-genomic form of MAPK inhibitor resistance mediates cross-resistance to anti-PD-1 therapy.

Single-cell RNA sequencing from melanoma tumors and computational analyses identified a resistance program expressed by malignant cells that is associated with T cell exclusion and immune evasion. This program is expressed prior to immunotherapy, characterizes cold niches in situ, and predicted clinical response to anti-PD-1 therapy in an independent cohort of melanoma patients. CDK4/6-inhibition represses this program in individual malignant cells, suggesting a new therapeutic strategy [46].

High-dimensional single-cell mass cytometry and a bioinformatics pipeline was used for the characterization of immune cell subsets in the peripheral blood of patients with advanced melanoma before and after 12 weeks of anti-PD-1 therapy [47]. During therapy, a clear response to immunotherapy was seen in the T cell compartment. However, before commencing therapy, a strong predictor of PFS and OS was the frequency of CD14+CD16−HLA-DRhi monocytes. Thus, the frequency of monocytes in peripheral blood mononuclear cells may support clinical decision-making.

Evaluation of > 300 patient samples across 22 tumor types from four KEYNOTE clinical trials of pembrolizumab identified both TMB and a T cell-inflamed gene expression profile as having predictive utility in identifying responders and non-responders to anti-PD-1 therapy [48]. TMB and gene expression profile were independently predictive of response and demonstrated low correlation, suggesting that they capture distinct features of neo-antigenicity and T cell activation. Immune gene expression signatures were also prognostic for response to combined BRAF/MEK inhibition [49]. High TMB added positive prognostic value to immune gene signatures in the placebo arm (high IFN-γ and high TMB associated with longer RFS), whereas IFN-γ gene signature identified patients with longer RFS independently of TMB status in the dabrafenib plus trametinib arm.

**Fig. 2:**
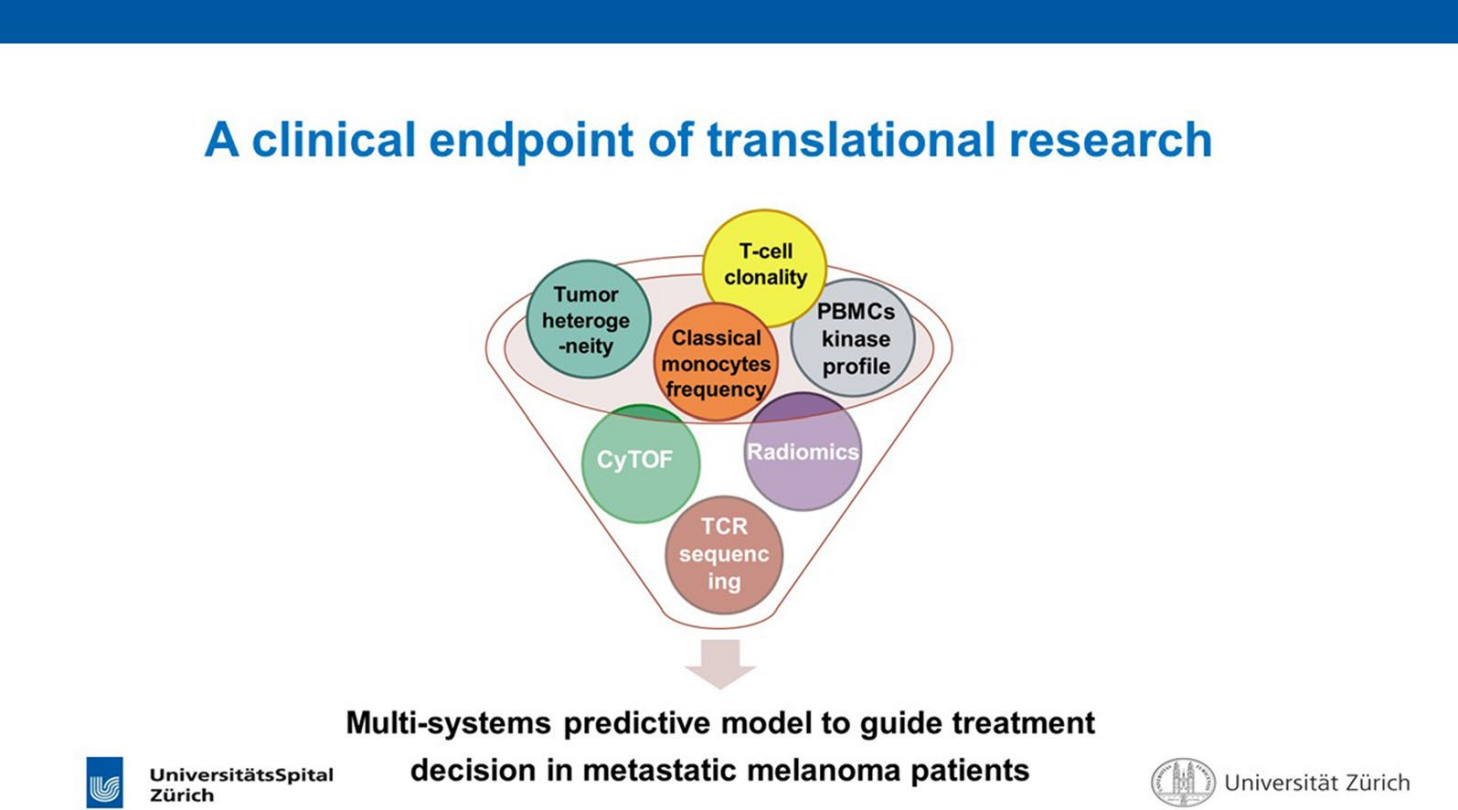
A clinical endpoint of translational research. A clinical goal of translational research is a multi-system predictive model to guide treatment decision-making in metastatic melanoma

These examples all indicate a fundamental role of translational research in helping guide therapy. A clinical goal of translational research is a multi-system predictive model to guide treatment decision-making in metastatic melanoma (Fig. 2).

#### Key points

- In addition to NGS, a multitude of other cutting-edge technologies are also involved in translational research, including single-cell mass cytometry/imaging mass cytometry, fast drug-testing, deep drug, genomics and transcriptomics.
- Many examples of translational research indicate its fundamental role in helping guide therapy.
- A clinical goal of translational research is a multi-system predictive model to guide treatment decision-making in metastatic melanoma.

### Role of adrenergic stress reversal in melanoma therapy

Adrenergic signalling promotes tumor growth and metastasis in many ways. Tumors are innervated by postganglionic nerves of the sympathetic nervous system and, in response to stress, these nerves secrete norepinephrine (NE). Many cells in the TME express adrenergic receptors, and their responses support tumor growth.

β-Adrenergic receptor signalling can influence immune cell function. NE signalling through β-adrenergic receptors on T cells is highly suppressive, having been shown to decrease antigen-specific CD8+ T cell frequency and functionality, impair CD8+ T cell killing, suppress CD8+ T cell production of IFN-γ and tumor necrosis factor (TNF)-α, decrease IL-2 production and proliferation of TH1 CD4+ T cells, and prevent T cell egress from lymph nodes. However, most of this work has been done in models of infection and autoimmunity, and its role in cancer has been largely unexplored.

The effects of physical/physiological environmental stressors on tumor growth have not been as well studied but environmental factors can induce a stress response and alter internal metabolism and physiology. Mice housed at an ambient temperature of 30 °C have significantly inhibited tumor growth compared to mice housed at standard temperatures (22 °C) even though mice under both conditions maintain a normal core body temperature of 37 °C [50]. Cold-stressed mice have elevated systemic levels of NE. Chronic adrenergic signalling in cold-stressed mice promotes tumor growth by induction of antiapoptotic signalling molecules and by profound immunosuppression of the antitumor immune response. Reducing thermal stress helps to control tumor growth with this effect dependent on the adaptive immune system.

Reducing adrenergic stress by physiologic reduction of NE signalling improved anti-PD-1 efficacy [51]. Moreover, combining β-blockade with anti-PD-1 therapy had a synergistic inhibitory effect on tumor growth while anti-PD-1 alone had no impact. The benefit of propranolol was lost in the absence of CD8+ T cells and propranolol improved the functional orientation of CD8+ TILs as evidenced by increased expression of markers of effector function.

The question is whether the sympathetic nervous system can function as a checkpoint and regulate anti-tumor immunity and response to cancer therapies? Retrospective observational data have suggested a significant survival benefit for melanoma patients prescribed pan β-blockers compared to either those taking no β-blocker or β1-selective blockers [52]. βAR blockade also enhanced control of murine melanoma growth by anti-PD-1 checkpoint blockade, with this effect most significant when a β-blocker was combined with dual anti-PD-1 and high-dose IL-2 therapy. Several other retrospective studies have also suggested that the incidental use of β-blockers is beneficial to cancer patients.

Based on these findings, a phase IB/II study of propranolol with fixed-dose pembrolizumab in patients with unresectable stage III and stage IV melanoma has been initiated.

#### Key points

- Adrenergic signalling promotes tumor growth and metastasis.
- Norepinephrine (NE) signalling through β-adrenergic receptors on T cells is highly immunosuppressive, but its role in cancer has been largely unexplored.
- In preclinical studies, reducing adrenergic stress by physiologic reduction of NE signalling improved anti-PD-1 efficacy, while combining β-blockade with anti-PD-1 therapy had a synergistic inhibitory effect on tumor growth while anti-PD-1 alone had no impact.
- Several retrospective studies have suggested that the incidental use of β-blockers is beneficial to cancer patients.
- Based on these findings, a phase IB/II study of propranolol with fixed-dose pembrolizumab in patients with unresectable stage III and stage IV melanoma has been initiated.

### Advancing the understanding and treatment of melanoma CNS metastases

Melanoma has the highest risk of brain metastases among solid tumors, occurring in 10–20% of patients at diagnosis of stage IV, up to 50% over the course of the disease and up to 70% at autopsy. Historically, median OS of patients with melanoma brain metastases has been approximately 4 months, with traditional blood–brain barrier-penetrating chemotherapies achieving intracranial responses in less than 10% of patients. Targeted agents and immunotherapies have demonstrated significant benefits in patients with metastatic disease but patients with active brain metastases have typically been excluded from large trials. Three recent studies in patients with melanoma brain metastases have provided promising results.

In the COMBI-MB trial, dabrafenib plus trametinib was assessed in patients with asymptomatic melanoma brain metastases [53]. After a median follow-up of 8.5 months, intracranial response rate was 58% in 76 patients with BRAFV600E-mutant disease and without prior local therapy. Similar response rates were also observed in smaller cohorts which varied by BRAF mutation, prior local therapy and whether disease was symptomatic or not. Median PFS was 5.6 months. Disease progression was intracranial only in 47% of patients. With regard to immune checkpoint inhibition, the efficacy and safety of nivolumab plus ipilimumab has been evaluated in the CheckMate 204 study, in which the rate of intracranial clinical benefit was 57% in 94 patients with melanoma metastatic to the brain [54]. Intracranial PFS rate was 59.5%, with extracranial PFS rate only slightly higher and OS rate was 81.5% at 12 months. Nivolumab plus ipilimumab (versus nivolumab alone) were also assessed in patients with melanoma brain metastases in the Anti-PD1 Brain Collaboration (ABC) study [55]. At a median follow up of 17 months, intracranial responses were achieved by 46% patients receiving nivolumab plus ipilimumab and 20% receiving nivolumab monotherapy. Six-month intracranial PFS rate was 53% with the combination and 20% with nivolumab.

Systemic therapy for melanoma brain metastases has offered marked progress but more research is needed. Combination therapy with ipilimumab plus nivolumab is better than single-agent PD-1 therapy and represents a new standard of care for patients with asymptomatic brain metastases not requiring steroids but 30–40% of patients have progressive disease, toxicity is an issue and there are no data for patients on steroids. Targeted therapy of combined BRAF and MEK inhibition offers rapid responses and initial disease control, including in patients on steroids. However, most responses are ≤ 6 months and around half of patients have intracranial before extracranial progression. Next steps involve further investigation of different combinatorial approaches (e.g. stereotactic radiosurgery with immune checkpoint inhibitors), further characterization of melanoma brain metastases to direct rational approaches, and more research into the treatment of symptomatic disease, patients with leptomeningeal disease, and treatment options after failure on ipilimumab plus nivolumab or dabrafenib plus trametinib.

#### Key points

- Targeted agents and immunotherapies have demonstrated significant benefits in patients with metastatic disease but patients with active brain metastases have typically been excluded from large trials.
- Three recent studies in patients with melanoma brain metastases have provided promising results (COMBI-MB, CheckMate 204 and the Anti-PD1 Brain Collaboration studies).
- Combination therapy with ipilimumab plus nivolumab is better than single-agent PD-1 therapy and represents a new standard of care for patients with asymptomatic brain metastases not requiring steroids.
- Targeted therapy of combined BRAF and MEK inhibition offers rapid responses and initial disease control, including in patients on steroids but most responses are ≤ 6 months and around half of patients have intracranial before extracranial progression.
- Next steps include further investigation of different combinatorial approaches, treatment options after failure on ipilimumab plus nivolumab or dabrafenib plus trametinib.

### STING agonism and type I interferon to augment immunotherapy

A T cell-inflamed TME may serve as a predictive biomarker for response to immunotherapies. Given this, how can we approach non-T cell-infiltrated tumors? Innate immune sensing of tumors is largely driven by the STING pathway. STING^−/−^ mice fail to generate an effective spontaneous antitumor T-cell response, which is associated with defective recruitment and activation of the Batf3-lineage DCs expressing CD8α or CD103 [56]. Downstream from STING pathway activation is the induction of type I IFNs which is critical for cross-presentation of antigens by Batf3-lineage DCs to CD8+ T cells.

STING agonists may provide a means to deliberately initiate innate immune inflammation to promote an endogenous T cell response in non-T cell-inflamed tumors. Intratumoral administration of the STING agonist, MK-1454, results in complete tumor regression and enhances the efficacy of anti-PD-1 therapy in mouse syngeneic models. In a phase 1 study, MK-1454 in combination with pembrolizumab had encouraging efficacy and an acceptable safety profile in patients with advanced solid tumors or lymphoma [57]. Elevations in serum cytokines IL-6 and IP-10 and STING induced gene expression in blood were observed. Another intratumoral STING agonist, MIW815, was well tolerated as a single-agent with no dose-limiting toxicities noted and signs of biological activity in patients with advanced solid tumors and lymphoma [58].

Stereotactic body radiotherapy (SBRT) may stimulate innate and adaptive immunity to augment response to immunotherapy. Multi-organ SBRT followed by pembrolizumab was shown to be well tolerated with acceptable toxicity in patients with metastatic solid tumors [17]. Up-regulated genes post-SBRT are enriched by pathways analysis for innate and adaptive immunity and DNA damage response regulation. Large partially irradiated tumors exhibit control similar to smaller completely irradiated tumors.

The first STING agonists are being tested but may require novel approaches to overcome intratumoral delivery. BMS-986301 is a more potent STING agonist for which intramuscular administration may be possible [59] while SB-11285 may allow intravenous or tumor-targeted STING agonism. Systemic inhibition of phosphodiesterase ENPP1 may decrease the threshold for STING agonism and TREX1 inhibitor administered via an infectious vector may offer a systemic indirect STING agonism approach. Tumor-targeted or systemic STING agonism may be the ideal partner for combination cancer therapeutic development.

#### Key points

- Given that a T cell-inflamed TME may serve as a predictive biomarker for response to immunotherapies, how do we treat non-T cell-infiltrated tumors?
- STING agonists may provide a means to deliberately initiate innate immune inflammation to promote an endogenous T cell response in non-T cell-inflamed tumors.
- Intratumoral administration of the STING agonist, MK-1454, resulted in complete tumor regression and enhances the efficacy of anti-PD-1 therapy in mouse syngeneic models and showed encouraging efficacy and an acceptable safety profile in combination with pembrolizumab in patients with advanced solid tumors or lymphoma.
- Tumor-targeted or systemic STING agonism may be the ideal partner for combination cancer therapeutic development.

## Conclusions

Since 2011, the use of novel immunotherapies and targeted agents have significantly improved outcomes for patients with advanced melanoma. The challenge for the IO field is to achieve even greater improvements through optimizing treatment regimens, which increasingly involve drug combinations. Importantly, development of biomarkers that allow for selection of patients that will benefit from treatment through better understanding of the interaction of the tumor and immune response is a critical need. The advent of new and improved standards of care is likely to continue and further enhance the long term survival of patients. Specifically, the use of immune checkpoint inhibitors and MAPK inhibitors is expanding from the metastatic setting to encompass adjuvant as well as neo-adjuvant therapies, with increasing evidence of benefits in these populations.

## Data Availability

Not applicable.

## Abbreviations

ctDNA: circulating tumor DNA
CSPG4: chondroitin sulphate peptidoglycan 4
CTLA-4: cytotoxic T-lymphocyte-associated antigen
CVA21: coxsackievirus A21
DC: dendritic cell
EFS: event-free survival
FMT: fecal microbiota transplantation
HR: hazard ratio
IFN: interferon
IL: interleukin
IPRES: innate anti-PD-1 resistance
LAG-3: lymphocyte-activating gene-3
LDH: lactate dehydrogenase
MAPK: mitogen-activated protein kinase
MTEX: melanoma-derived exosomes
NCSC: neural crest stem cell
NE: norepinephrine
NGS: next generation sequencing
NSCLC: non-small-cell lung cancer
ORR: objective response rate
OS: overall survival
pCR: pathological complete response
PD-1: programmed death-1
PD-L1: programmed death ligand-1
PFS: progression-free survival
RCC: renal cell carcinoma
RFS: relapse-free survival
SBRT: stereotactic body radiotherapy
SEC: size exclusion chromatography
SLD: sum of longest diameters of target lesions
TEX: tumor-derived exosomes
TILs: tumor-infiltrating lymphocytes
TMB: tumor mutational burden
TME: tumor microenvironment
TNF: tumor necrosis factor
TVEC: talimogene laherparepvec
WES: whole exome sequencing
WGS: whole genome sequencing

## References

[1] Eggermont AM, Chiarion-Sileni V, Grob JJ, Dummer R, Wolchok JD, Schmidt H (2016). Prolonged survival in stage III melanoma with ipilimumab adjuvant therapy. N Engl J Med.

[2] Tarhini AA, Lee SJ, Li X, Rao UNM, Nagarajan A, Albertini MR (2018). E3611—a randomized phase II study of ipilimumab at 3 or 10 mg/kg alone or in combination with high-dose interferon-α2b in advanced melanoma. Clin Cancer Res.

[3] Weber J, Mandala M, Del Vecchio M, Gogas HJ, Arance AM, Cowey CL (2017). Adjuvant nivolumab versus ipilimumab in resected stage III or IV melanoma. N Engl J Med.

[4] Eggermont AMM, Blank CU, Mandala M, Long GV, Atkinson V, Dalle S (2018). Adjuvant pembrolizumab versus placebo in resected stage III melanoma. N Engl J Med.

[5] Long GV, Hauschild A, Santinami M, Atkinson V, Mandalà M, Chiarion-Sileni V (2017). Adjuvant dabrafenib plus trametinib in stage III BRAF-mutated melanoma. N Engl J Med.

[6] Amaria RN, Reddy SM, Tawbi HA, Davies MA, Ross MI, Glitza IC (2018). Neoadjuvant immune checkpoint blockade in high-risk resectable melanoma. Nat Med.

[7] Blank CU, Rozeman EA, Fanchi LF, Sikorska K, van de Wiel B, Kvistborg P (2018). Neoadjuvant versus adjuvant ipilimumab plus nivolumab in macroscopic stage III melanoma. Nat Med.

[8] Blank CU, Rozeman EA, Menzies AM, van de Wiel BA, Adhikari C, Sikorska K (2018). OpACIN-neo: a multicenter phase II study to identify the optimal neo-adjuvant combination scheme of ipilimumab (IPI) and nivolumab (NIVO). Ann Oncol.

[9] Chan TA, Yarchoan M, Jaffee E, Swanton C, Quezada SA, Stenzinger A (2018). Development of tumor mutation burden as an immunotherapy biomarker: utility for the oncology clinic. Ann Oncol.

[10] Goodman AM, Kato S, Bazhenova L, Patel SP, Frampton GM, Miller V (2017). Tumor mutational burden as an independent predictor of response to immunotherapy in diverse cancers. Mol Cancer Ther.

[11] Lee JH, Long GV, Boyd S, Lo S, Menzies AM, Tembe V (2017). Circulating tumour DNA predicts response to anti-PD1 antibodies in metastatic melanoma. Ann Oncol.

[12] Herbreteau G, Vallée A, Knol AC, Théoleyre S, Quéreux G, Varey E (2018). Quantitative monitoring of circulating tumor DNA predicts response of cutaneous metastatic melanoma to anti-PD1 immunotherapy. Oncotarget.

[13] Dummer R, Schadendorf D, Ascierto PA, Arance A, Dutriaux C, Di Giacomo AM (2017). Binimetinib versus dacarbazine in patients with advanced NRAS-mutant melanoma (NEMO): a multicentre, open-label, randomised, phase 3 trial. Lancet Oncol.

[14] Hong A, Moriceau G, Sun L, Lomeli S, Piva M, Damoiseaux R (2018). Exploiting drug addiction mechanisms to select against MAPKi-resistant melanoma. Cancer Discov.

[15] Demaria S, Kawashima N, Yang AM, Devitt ML, Babb JS, Allison JP (2005). Immune-mediated inhibition of metastases after treatment with local radiation and CTLA-4 blockade in a mouse model of breast cancer. Clin Cancer Res.

[16] Vanpouille-Box C, Alard A, Aryankalayil MJ, Sarfraz Y, Diamond JM, Schneider RJ (2017). DNA exonuclease Trex1 regulates radiotherapy-induced tumour immunogenicity. Nat Commun.

[17] Luke JJ, Lemons JM, Karrison TG, Pitroda SP, Melotek JM, Zha Y (2018). Safety and clinical activity of pembrolizumab and multisite stereotactic body radiotherapy in patients with advanced solid tumors. J Clin Oncol.

[18] Theelen W, Peulen H, Lalezari F, de Vries J, De Langen J, Aerts J (2018). Randomized phase II study of pembrolizumab after stereotactic body radiotherapy (SBRT) versus pembrolizumab alone in patients with advanced non-small cell lung cancer: the PEMBRO-RT study. J Clin Oncol.

[19] Antonia SJ, Villegas A, Daniel D, Vicente D, Murakami S, Hui R (2017). Durvalumab after chemoradiotherapy in stage III non-small-cell lung cancer. N Engl J Med.

[20] Shaverdian N, Lisberg AE, Bornazyan K, Veruttipong D, Goldman JW, Formenti SC (2017). Previous radiotherapy and the clinical activity and toxicity of pembrolizumab in the treatment of non-small-cell lung cancer: a secondary analysis of the KEYNOTE-001 phase 1 trial. Lancet Oncol.

[21] Formenti SC, Rudqvist NP, Golden E, Cooper B, Wennerberg E, Lhuillier C (2018). Radiotherapy induces responses of lung cancer to CTLA-4 blockade. Nat Med.

[22] Daud A, Pavlick AC, Ribas A, Gonzalez R, Lewis KD, Hamid O, et al. Extended follow‐up results of a phase 1b study (BRIM7) of cobimetinib (C) combined with vemurafenib (V) in BRAFV600‐mutated melanoma. In: Presented at the 14th international congress of the society for melanoma research/9th world congress of melanoma; October 18–21, 2017; Brisbane, Queensland, Australia.

[23] Hauschild A, Larkin J, Ribas A, Dréno B, Flaherty KT, Ascierto PA (2018). Modeled prognostic subgroups for survival and treatment outcomes in BRAF V600-mutated metastatic melanoma: pooled analysis of 4 randomized clinical trials. JAMA Oncol.

[24] Schadendorf D, Long GV, Stroiakovski D, Karaszewska B, Hauschild A, Levchenko E (2017). Three-year pooled analysis of factors associated with clinical outcomes across dabrafenib and trametinib combination therapy phase 3 randomised trials. Eur J Cancer.

[25] Massi D, Brusa D, Merelli B, Falcone C, Xue G, Carobbio A (2015). The status of PD-L1 and tumor-infiltrating immune cells predict resistance and poor prognosis in BRAFi-treated melanoma patients harboring mutant BRAFV600. Ann Oncol.

[26] Sullivan RJ, Gonzalez R, Lewis KD, Hamid O, Infante JR, Patel MR (2017). Atezolizumab (A) + cobimetinib (C) + vemurafenib (V) in BRAFV600-mutant metastatic melanoma (mel): updated safety and clinical activity. J Clin Oncol.

[27] Aspeslagh S, Morel D, Soria JC, Postel-Vinay S (2018). Epigenetic modifiers as new immunomodulatory therapies in solid tumours. Ann Oncol.

[28] Agarwala SS, Moschos SJ, Johnson ML, Opyrchal M, Gabrilovich D, Danaher P (2018). Efficacy and safety of entinostat (ENT) and pembrolizumab (PEMBRO) in patients with melanoma progressing on or after a PD-1/L1 blocking antibody. J Clin Oncol.

[29] Hong CS, Funk S, Muller L, Boyiadzis M, Whiteside TL (2016). Isolation of biologically active and morphologically intact exosomes from plasma of patients with cancer. J Extracell Vesicles.

[30] Sharma P, Ludwig S, Muller L, Hong CS, Kirkwood JM, Ferrone S (2018). Immunoaffinity-based isolation of melanoma cell-derived exosomes from plasma of patients with melanoma. J Extracell Vesicles.

[31] Vétizou M, Pitt JM, Daillère R, Lepage P, Waldschmitt N, Flament C (2015). Anticancer immunotherapy by CTLA-4 blockade relies on the gut microbiota. Science.

[32] Routy B, Le Chatelier E, Derosa L, Duong CPM, Alou MT, Daillère R (2018). Gut microbiome influences efficacy of PD-1-based immunotherapy against epithelial tumors. Science.

[33] Derosa L, Hellmann MD, Spaziano M, Halpenny D, Fidelle M, Rizvi H (2018). Negative association of antibiotics on clinical activity of immune checkpoint inhibitors in patients with advanced renal cell and non-small-cell lung cancer. Ann Oncol.

[34] Ayers M, Lunceford J, Nebozhyn M, Murphy E, Loboda A, Kaufman DR (2017). IFN-γ-related mRNA profile predicts clinical response to PD-1 blockade. J Clin Invest.

[35] Horton BL, Williams JB, Cabanov A, Spranger S, Gajewski TF (2017). Intratumoral CD8+ T-cell apoptosis is a major component of T cell dysfunction and impedes anti-tumor immunity. Cancer Immunol Res.

[36] Spranger S, Dai D, Horton B, Gajewski TF (2017). Tumor-residing Batf3 dendritic cells are required for effector T cell trafficking and adoptive T cell therapy. Cancer Cell.

[37] Puzanov I, Milhem MM, Minor D, Hamid O, Li A, Chen L (2016). Talimogene laherparepvec in combination with ipilimumab in previously untreated, unresectable stage IIIB–IV melanoma. J Clin Oncol.

[38] Chesney J, Awasthi S, Curti B, Hutchins L, Linette G, Triozzi P (2018). Phase IIIb safety results from an expanded-access protocol of talimogene laherparepvec for patients with unresected, stage IIIB–IVM1c melanoma. Melanoma Res.

[39] Andtbacka RHI, Ross MI, Agarwala SS, Taylor MH, Vetto JT, Neves RI (2017). Final results of a phase II multicenter trial of HF10, a replication-competent HSV-1 oncolytic virus, and ipilimumab combination treatment in patients with stage IIIB–IV unresectable or metastatic melanoma. J Clin Oncol.

[40] Curti BD, Richards JM, Hallmeyer S, Faries MB, Andtbacka RHI, Daniels GA (2017). Activity of a novel immunotherapy combination of intralesional Coxsackievirus A21 and systemic ipilimumab in advanced melanoma patients previously treated with anti-PD1 blockade therapy. J Clin Oncol.

[41] Agarwala SS, Ross M, Zager J, Shirai K, Essner R, Smithers BM, et al. Interim results of a phase 1b/2 study of PV-10 and PD-1 blockade in advanced melanoma. In: Presented at the 15th international congress of the society for melanoma research, Manchester, UK, October 24–27, 2018.

[42] Algazi AP, Tsai KT, Rosenblum M, Fox BA, Andtbacka RHI, Li A, et al. Immune monitoring outcomes of patients with stage III/IV melanoma treated with a combination of pembrolizumab and intratumoral plasmid interleukin 12 (pIL-12). J Clin Oncol 2017;35(suppl 7S; abstract 78).

[43] Daud AI, Loo K, Pauli ML, Sanchez-Rodriguez R, Sandoval PM, Taravati K (2016). Tumor immune profiling predicts response to anti-PD-1 therapy in human melanoma. J Clin Invest.

[44] Rambow F, Rogiers A, Marin-Bejar O, Aibar S, Femel J, Dewaele M (2018). Toward minimal residual disease-directed therapy in melanoma. Cell.

[45] Hugo W, Zaretsky JM, Sun L, Song C, Moreno BH, Hu-Lieskovan S (2016). Genomic and transcriptomic features of response to anti-PD-1 therapy in metastatic melanoma. Cell.

[46] Jerby-Arnon L, Shah P, Cuoco MS, Rodman C, Su MJ, Melms JC (2018). A cancer cell program promotes T cell exclusion and resistance to checkpoint blockade. Cell.

[47] Krieg C, Nowicka M, Guglietta S, Schindler S, Hartmann FJ, Weber LM (2018). High-dimensional single-cell analysis predicts response to anti-PD-1 immunotherapy. Nat Med.

[48] Cristescu R, Mogg R, Ayers M, Albright A, Murphy E, Yearley J (2018). Pan-tumor genomic biomarkers for PD-1 checkpoint blockade-based immunotherapy. Science.

[49] Long GV, Hauschild A, Santinami M, Atkinson V, Mandalà M, Chiarion-Sileni V (2018). Updated relapse-free survival (RFS) and biomarker analysis in the COMBI-AD trial of adjuvant dabrafenib + trametinib (D + T) in patients (pts) with resected BRAF 600–mutant stage III melanoma. Ann Oncol.

[50] Kokolus KM, Capitano ML, Lee CT, Eng JW, Waight JD, Hylander BL (2013). Baseline tumor growth and immune control in laboratory mice are significantly influenced by subthermoneutral housing temperature. Proc Natl Acad Sci USA.

[51] Bucsek MJ, Qiao G, MacDonald CR, Giridharan T, Evans L, Niedzwecki B (2017). β-Adrenergic signaling in mice housed at standard temperatures suppresses an effector phenotype in CD8+ T cells and undermines checkpoint inhibitor therapy. Cancer Res.

[52] Kokolus KM, Zhang Y, Sivik JM, Schmeck C, Zhu J, Repasky EA (2017). Beta blocker use correlates with better overall survival in metastatic melanoma patients and improves the efficacy of immunotherapies in mice. Oncoimmunology.

[53] Davies MA, Saiag P, Robert C, Grob JJ, Flaherty KT, Arance A (2017). Dabrafenib plus trametinib in patients with BRAFV600-mutant melanoma brain metastases (COMBI-MB): a multicentre, multicohort, open-label, phase 2 trial. Lancet Oncol.

[54] Tawbi HA, Forsyth PA, Algazi A, Hamid O, Hodi FS, Moschos SJ (2018). Combined nivolumab and ipilimumab in melanoma metastatic to the brain. N Engl J Med.

[55] Long GV, Atkinson V, Lo S, Sandhu S, Guminski AD, Brown MP (2018). Combination nivolumab and ipilimumab or nivolumab alone in melanoma brain metastases: a multicentre randomised phase 2 study. Lancet Oncol.

[56] Woo SR, Fuertes MB, Corrales L, Spranger S, Furdyna MJ, Leung MY (2014). STING-dependent cytosolic DNA sensing mediates innate immune recognition of immunogenic tumors. Immunity.

[57] Harrington KJ, Brody J, Ingham M, Strauss J, Cemerski S, Wang M (2018). Preliminary results of the first-in-human (FIH) study of MK-1454, an agonist of stimulator of interferon genes (STING), as monotherapy or in combination with pembrolizumab (pembro) in patients with advanced solid tumors or lymphomas. Ann Oncol.

[58] Meric-Bernstam F, Werner T, Hodi S, Messersmith W, Lewis N, Talluto C, et al. Phase I dose-finding study of MIW815 (ADU-S100), an intratumoral STING agonist, in patients with advanced solid tumors or lymphomas. SITC; 2018.

[59] Schieven G, Brown J, Swanson J, Stromko BSC, Ho C-P, Zhang R, et al Preclinical characterization of BMS-986301, a differentiated STING agonist with robust antitumor activity as monotherapy or in combination with anti-PD-1. SITC; 2018.

[60] Weber J, Mandala M, Del Vecchio M, et al. Adjuvant therapy with nivolumab (NIVO) versus ipilimumab (IPI) after complete resection of stage III/IV melanoma: updated results from a phase III trial (CheckMate 238). In: Presented at American society of clinical oncology 2018 annual meeting; June 4, 2018; Chicago, IL.

[61] Hauschild A, Santinami M, Long G, et al. Adjuvant dabrafenib (D) plus trametinib (T) for resected stage III BRAF V600E/K-mutant melanoma. In: Presented at European society for medical oncology 2017 congress; September 11, 2017; Madrid, Spain.

